# The effects of intradialytic resistance training on muscle strength, psychological well-being, clinical outcomes and circulatory micro-ribonucleic acid profiles in haemodialysis patients: Protocol for a quasi-experimental study

**DOI:** 10.1097/MD.0000000000015570

**Published:** 2019-05-13

**Authors:** Aurel Zelko, Ivana Skoumalova, Peter Kolarcik, Jaroslav Rosenberger, Miroslava Rabajdova, Maria Marekova, Andrea Madarasova Geckova, Jitse P. van Dijk, Sijmen A. Reijneveld

**Affiliations:** a Institute of Physical Education and Sport, Pavol Jozef Safarik University, Kosice, Slovakia; b Graduate School Kosice Institute for Society and Health, Faculty of Medicine, Pavol Jozef Safarik University, Kosice, Slovakia; c Department of Health Psychology, Faculty of Medicine, Pavol Jozef Safarik University, Kosice, Slovakia; d Department of Community and Occupational Medicine, University Medical Center Groningen, University of Groningen, Hanzeplein, Groningen, Netherlands; e Olomouc University Society and Health Institute, Palacky University Olomouc, Olomouc, Czech Republic; f 2nd Department of Internal Medicine, Faculty of Medicine, Pavol Jozef Safarik University, Kosice, Slovakia; g Fresenius Medical Care - Dialysis Services Kosice, Kosice, Slovakia; h Department of Medical and Clinical Biochemistry, Faculty of Medicine, Pavol Jozef Safarik University, Kosice, Slovakia; i NEPHRO-team, Faculty of Medicine, Pavol Jozef Safarik University, Kosice, Slovakia.

**Keywords:** anxiety, depression, haemodialysis therapy, health literacy, miRNA, muscle strength, quality of life, resistance training

## Abstract

**Background:**

Intradialytic resistance training (IRT) protects patients’ muscle mass and functions against protein-energy wasting, malnutrition and cachexia. However, the evidence of the effects of such an intervention in haemodialysis patients is limited and not conclusive. To improve the applicability of such interventions, we need a better understanding of molecular, functional and psycho-social adaptation in dialysed patients following a physical training. Therefore, the aim of this study is to investigate the effects of IRT on lower extremity muscle functions, quality of life, and anxiety and depression, clinical outcomes and circulatory micro-ribonucleic acid (miRNA) profiles in patients on chronic haemodialysis therapy.

**Methods:**

We will perform a quasi-experimental study in 3 dialysis centres. Patients will be recruited via their nephrologists and will be allocated to an experimental and a control group based on the location of the patients’ dialysis centre. Patients allocated to the experimental group will undergo a 12-week IRT, while the control group will remain physically inactive during dialysis. The primary outcome is the change in the maximal force produced during an isometric contraction of lower extremity muscles. Secondary outcomes regard quality of life, anxiety and depression, clinical outcomes and circulatory miRNA profiles. Patients’ level of health literacy defined as the ability to get and understand health information will be also measured in the study as a potential modifier of effects.

**Discussion:**

This quasi-experimental study can add in an important way to our understanding of the effects of resistance training on dialysis patients’ muscle strength, quality of life and disease-specific outcomes.

## 1 Introduction

Chronic kidney disease stage 5 (CKD-5) is a major health problem globally with a significant increase of its socio-economic burden anticipated in the future. Based on the analysis of observational studies and renal therapy registries in the year 2010, more than 2.6 million people were receiving renal replacement therapy (RRT) worldwide. The projection of the prevalence of CKD-5 for the year 2030 indicated a nearly double increase in RRT receiver's number across the world population. Based on the demographical projection realized by analysis of 35 national renal registries (covering 98% of the European renal registries), for the European countries the number of people receiving RRT will raise from 0.532 million in 2010 to 0.825 million in 2030.^[1]^ This estimated trend in the prevalence of CKD-5 is increasing in particular due to an ageing of European population,^[2]^ and the increasing prevalence of diabetes and obesity.^[3,4]^

Patients diagnosed with chronic kidney disease require a nephrological monitoring of their kidney function and a therapy that reflects a patient's diagnosis and prognosis. Additionally to the therapy that affects the kidney functions, patients may have considered secondary prevention of CKD-5, which includes changes in physical activity behaviour. In particular, if a conservative, non-dialytic therapy is insufficient on the management of chronic kidney disease, the patient is submitted on regular haemodialysis therapy (HT).^[5]^ In patients with CKD-5 under 65 years of age, HT should improve mean life expectancy with over 10 years.^[6,7]^ The HT and the presence of different comorbidities are a source of several negative changes in the patient's physical activity behaviour.

The HT strongly contributes to an increase of patient's frailty^[8–10]^ and a decrease of the patient's muscle mass,^[11,12]^ functions,^[13,14]^ mobility,^[15]^ and independency.^[16]^ In the 1st months of the dialysis therapy, the inflammation^[17,18]^ and the protein-energy wasting^[19,20]^ generate metabolic complications that starts and accelerates the patient's sarcopenic obesity. Later on, the major HT side-effects are directly connected with regular exposition to prolonged inactivity due to this therapy.^[21,22]^ The HT requires patients in a stable sitting or supine position from 4 to 5 hours, with little or no physical activity, for 3 times a week. Regular inactivity in the patient's daytime for more than 3 hours is a behavioural risk factor and a strong determinant of further needs of hospitalization, specialized care, and prevalence of other comorbidities.^[23]^ Moreover, the presence of dialysis access (artery-vein fistula, graft or catheter), plus dietary and food restrictions reduce the number of physical activities suitable and safe for the patients on HT. These negative changes in patient's behaviour contributed to a decrease of muscle functions of upper and lower extremity muscles.^[15,14,24]^ To minimize these negative effects, the implementation of a specific exercise programme during dialysis is strongly desirable. Resistance training is considered as effective method in the prevention of muscle functional loss among dialysed patients.^[25]^ Therefore, our study is to analyse the effects of intradialytic resistance training (IRT) on the patient's lower extremity muscle functions.

In addition, patient's psychological outcomes are also negatively affected by HT and might to contribute to the patient's inactivity behaviour. Compared to the healthy population, patients on HT more frequently suffer from severe and persistent depressions.^[26]^ Under-diagnosed and untreated depressions in HT patients are associated with significantly higher relative risks of death, hospitalization and dialysis withdrawal.^[27]^ The presence of anxiety, emotional problems, and emotive coping style are also often prevalent in HT patients.^[28–30]^ The patients diagnosed with depression and anxiety disorders are less physically active compared to healthy controls^[31]^ and have a large amount of sedentary time and insufficient fulfilment of physical activity recommendations.^[32]^ The depression and anxiety may modify the patient's engagement and the effects of physical activity interventions performed during dialysis.^[33]^ Reversible, if the resistance training is well accepted by the patient, the depression and anxiety could be suppressed effectively.^[34,35]^ Moreover, the exercise performed during dialysis could significantly improve the patient's quality of life.^[36,37]^ Well-designed training intervention programs, evaluating the effects of IRT on patient's depression, anxiety, and quality of life in clinical care are rare and have not been adequately evaluated regarding contents and effects. To contribute to this area, we are analysing the effects of IRT on the prevalence of the anxiety and depression and quality of life among dialysed patients.

Adequate and safe physical activity lower all-caused and infection-related mortality and cause a better prognosis among dialysed patients.^[38–41]^ Given the exposition of IRT aimed at improving of patient's physical functions, there is a need for evaluation of training effects on specific renal outcomes. The existing evidence of clinical benefits following exercise among dialysed patients is highly conflicting. On the one hand, the literature is indicating positive effects on patient's dialysis adequacy measures, body composition and haematology profile.^[42–48]^ On the other hand, we are detecting studies that disapprove the impact of exercise on patient's clinical outcomes.^[49–55]^ Our current understanding of the training-induced therapeutic benefits is mostly originating from studies with flawed research designs. For atonement of contradictions in the evidence, we will assess the effect of IRT on patient's clinical outcomes.

Resistance training has been shown to modulate the micro-ribonucleic acid (miRNA) expression profiles among healthy sedentary and regularly exercised population.^[56–60]^ However, we did not find a conclusive body of evidence on the effects of resistance training on miRNA expression profile of patients with serious comorbidities. The only available trial that evaluated changes of the miRNAs during exercise intervention in patients with chronic kidney disease concluded no significant change of any studied miRNAs in exercised subjects compared with the usual care subjects.^[61]^ In that trial, Craenenbroeck et al were analyzing miRNAs involved in inflammation, apoptosis, hypoxia, ischemia adaptation, angiogenesis, hematopoiesis, progenitor cell mobilization, proliferation, and vascular calcification processes. For expansion and verification of the knowledge in the area of miRNA's role in adaptation processes of chronic kidney disease patients, we will analyze the effect of IRT on miRNAs involved in calcium metabolism, on miR206 involved in Insulin-like growth factor 1 (IGF-1) signalling pathway and on miR23-a involved in E3 ubiquitin-protein ligase (Tripartite motif containing 63 – TRIM63) signalling pathway. Also, proteins encoded of these signalling pathways will be analysed.

To sum up, evidence is scarce or lacking when it comes to the effects of IRT on the patient's physical functions, on the psycho-social variables, on the clinical and circulatory miRNA profiles. Therefore, we initiated a study to investigate the effects of IRT on the lower extremities muscular strength, on the quality of life, on the prevalence of anxiety and depression, on the clinical outcomes and on the circulatory miRNA expression profiles in HT receiving patients.

## 2 Aim of the study

The aim of this study is to investigate the effects of IRT on lower extremity muscle strength, on quality of life, anxiety and depression, on clinical outcomes and on circulatory miRNA profiles of patients on chronic HT.

## 3 Methods

### 3.1 Study design

We designed our research as a multicentre, quasi-experimental, parallel, 2 arm studies. To ensure sufficient replicability of our study, we describe its design following the Standard Protocol Items for Randomized Interventional Trials 2013 (SPIRIT 2013) statement,^[62]^ and made a schedule of enrolment, interventions and assessments planned on the study based on the SPIRIT 2013 template examples (Table 1).^[63]^ The study design and protocol were reviewed and approved by the Ethics Committee of the Pavol Jozef Safarik University in Kosice (approval no. 14N/2017); the protocol was registered at ClinicalTrials.gov (ID:NCT03511924). Prior to a patient's participation in the study, a written informed consent form will be signed and personally dated by the patient.

**Table 1:**
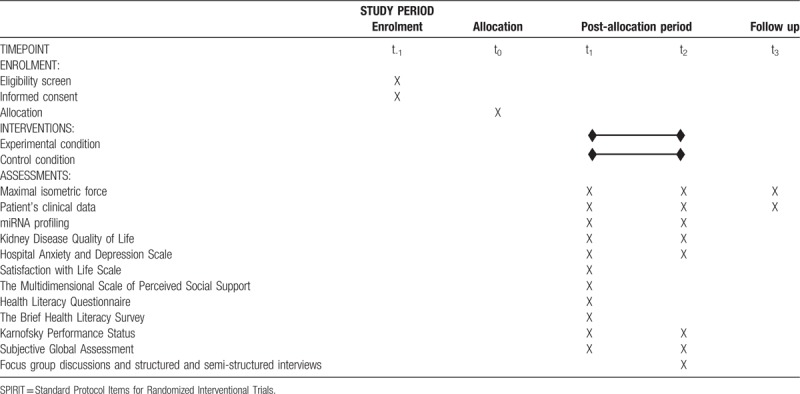
SPIRIT schedule describing the timeline of participant's enrolment process, application of interventions and performance of assessments planned for the experimental and control group in the study.

### 3.2 Eligibility criteria

All potentially eligible patients from 3 cooperating private dialysis centres (Fresenius Medical Care – Dialysis Services, and Logman East both located in Kosice; and in the Fresenius Medical Care – Dialysis Services located in Banska Bystrica) were screened and selected according to the inclusion and exclusion criteria through their nephrologists. The inclusion and exclusion criteria used during the selection of patients are summarized in Table 2.

**Table 2:**
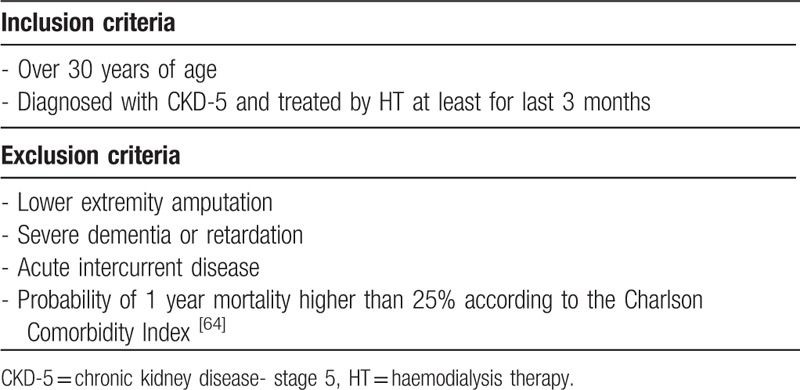
Inclusion and exclusion criteria applied during eligibility screen of patients in study.

### 3.3 Study participants

Eligible patients received oral information about the possibility to participate in the study through their nephrologists during regular nephrological consultations. Within a week, the research assistant personally contacted them during the dialysis session, and provided them with additional details about the study; they showed their availability to participate in the study. If the patient agreed to participate in the study, the research assistant acquainted the patient with a written informed consent form and the patient validated his/her voluntary agreement by a signature of consent. If the patient expressed disagreement with participation in the study, the research assistant collected the reasons for the patient's decline.

### 3.4 Sample size calculation

The study has been designed to detect an effect size of 0.60 for the intervention under study compared to control condition, regarding the primary outcome of change in the maximal voluntary force measured during isometric contraction of knee extensors, at an α level of 0.05 (2-tailed). Applying these sample size calculation parameters and previously reported changes in muscle strength during the resistance training intervention among dialysed patients,^[65]^ the study statistician designed a simulation to derive the required sample size. Simulation has indicated that 27 patients needed to be present in every arm of the study to detect a mean intervention mean difference in the maximal isometric force during the lower extremity muscles contraction, with 80% power. Based on studies with a similar design and primary outcome, we are expecting a 70% completion rate in the study.^[66,67]^ Therefore, the total number of patients minimally needed to be enrolled in the study is 78.

### 3.5 Allocation of participants

**Figure 1:**
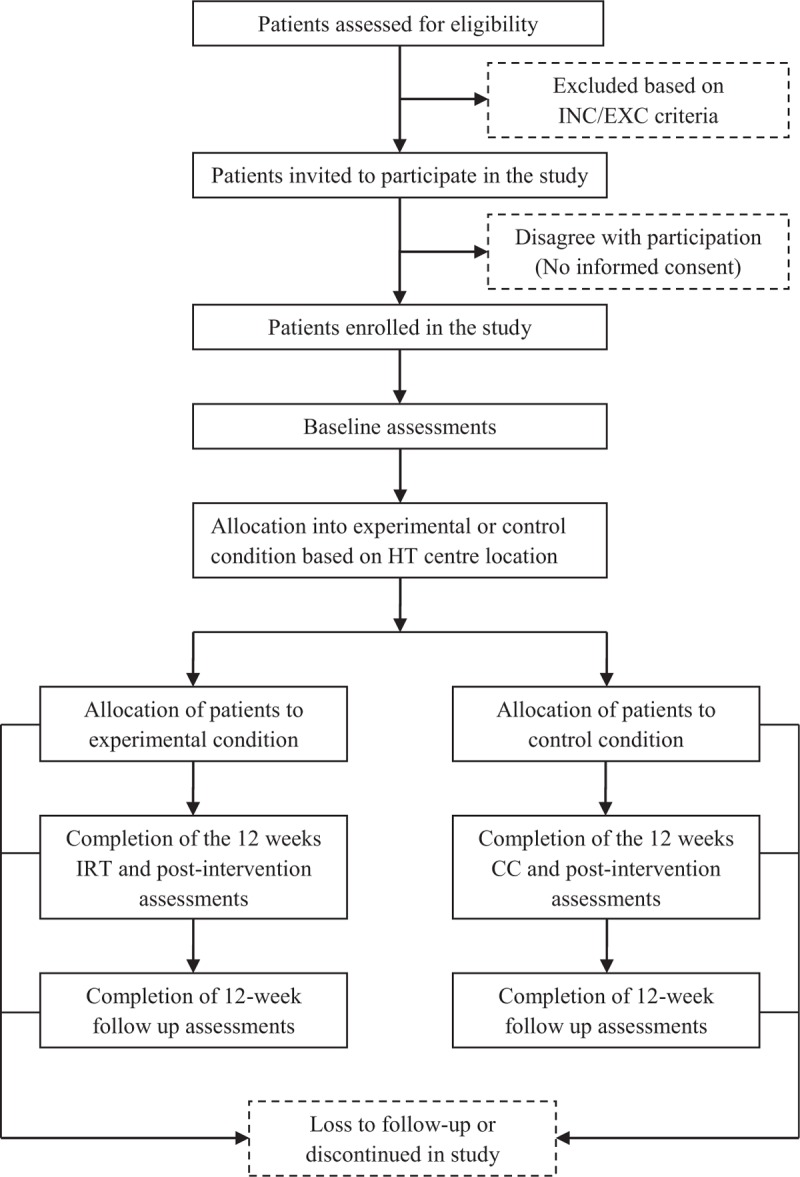
The flow chart of patients summarizing their eligibility assessment, enrolment and allocation into experimental and control group of the study. CC = control condition, INC/EXC = inclusion and exclusion, IRT = intradialytic resistance training.

Participants will be allocated into the experimental or control group based on the location of dialysis centre where they received HT. Patients attending the Fresenius Medical Care dialysis centre in Kosice and the Logman East dialysis centre in Kosice will be allocated into the experimental group; while patients from the Fresenius Medical Care dialysis centre located in Banska Bystrica will join the control group (Fig. 1).

### 3.6 Interventions

#### 3.6.1 Experimental condition

The experimental condition consisted of an IRT programme, performed under the supervision of training assistants, 3 sessions of 30-minutes per week, for 12 weeks. The exercise intervention will start a week after the completion of baseline assessments. All training sessions will be conducted during the HT, directly after safe connection of the patient to the dialysis unit and the time needed for serving a meal. Every session of training will start after the confirmation of the proper conditions from medical personnel of dialysis centre. During the training, each patient will be supervised and supported by 1 training assistant from the study investigatory team (NEPHRO team). This assistant is responsible for the evidence, control and management of the training session. If an assistant will notice any elevation of health risks or complications of the patient manifested during the training, it is the assistant's responsibility to adjust or terminate the actual training and report the situation. Training assistants for the IRT are experienced and specially trained for their positions in the implementation of the programme.

The IRT programme is designed to provide a sufficient stimulus for positive adaptations of neuromuscular systems while maximising safety, compliance and retention rates during the study. The IRT sessions will start with a 3-minute warm up and will be ended with a 7-minute cool-down and stretching. To perform effective exercises on supine position, we will use external pressure generated by elastic bands and over-balls (TheraBand, Akron, OH). These external loading resources will be fixed on a construction of the dialysis bed and during exercises patients will pull or push against them. In the setting of our study, elastic bands and over-balls are an effective source of external load and they both showed excellent applicability for implementation of IRT programmes in similar studies.^[65,68,69]^

The IRT programme will be tailored to each patient's abilities. The programme will include 3 exercises (unilateral push and pull of over-ball against a leg board, bilateral knee squeeze of over ball, and unilateral straight leg raise against the band pressure) that will enable tolerable activation of muscles executing knee extension, knee flexion, hip flexion, hip extension, hip abduction and hip adduction in supine position of the patient. During the first 2 weeks of the IRT programme, patients will perform per visit 3 sets (12 up to 15 repetitions each) of 3 different exercises of lower extremity muscles. Patients will take 1 to 2 minutes rest between each set. Once a patient will be capable to safely complete the planned programme for the actual session, the numbers of repetitions on the next session will increase with 3 repetitions for each exercise. If a patient reaches the maximal number of repetitions per exercise (18 repetitions) the assistant will increase the number of sets for one set. When the patient will be able to perform 5 sets of the 18 repetitions for each exercise, the assistant will make the resistance training harder by the application of a stiffer elastic band or over-ball with higher hardness. Vice versa, if a patient fails to complete the entire training session, or the supervisor notices obvious difficulties, the resistance will be facilitated by lowering the number of repetitions, sets or application of softer elastic bands and over-balls. Training assistants will register the adherence rates and fulfilment of the training plan in a paper training diary.

To control the patient's training progress, we will register the number of repetitions and series for each of exercise independently. For the registration of training effects on the patient, we will register delayed onset muscle soreness and knee joint range of motion. Construction and organization of the IRT programme will follow the recommendations published by the Exercise & Sports Science Australia.^[70]^

#### 3.6.2 Control condition

Patients allocated to the control group will receive their standard nephrological care. Through the 12-week period, all control participants will be instructed to maintain the standard treatment regimen and to maintain their customary dietary and physical activity patterns.

#### 3.6.3 Follow-up period

All patients enrolled in the study will undergo a 12 weeks follow-up period after the completion of the experimental or control condition. During the follow-up, the patients won’t be involved in any organized physical activity during the dialysis and all will return to their original HT regimen.

### 3.7 Outcomes

The primary outcome of the study is a change of maximal isometric force generated during the contraction of lower extremity muscles. The secondary outcomes of study which includes changes in the quality of life and prevalence of anxiety and depression, the renal specific clinical data, and the circulatory miRNA profile. The other background variables considered as possible modifiers and cofounders of training effects, are: patient's satisfaction with life, social support, health literacy, functional impairment and nutritional status. These variables are further specified in the following sections.

### 3.8 Primary and secondary outcomes assessments

#### 3.8.1 Primary outcome: procedure of muscle strength assessments

The assessments of the maximal isometric force generated during the contraction of lower extremity muscles will be realized in patients before and after the experimental or control condition and also after the 3 months follow-up period. The measurements will be realized according to a testing protocol for lower extremity muscle strength among dialysed patients published by Matsuzawa et al.^[71]^ Familiarization with the protocol on each test will be realized before the test procedures will take place. During the familiarization session, the investigator will explain the procedures of the test protocols to the patient and will acquire patient with proper execution of muscle contractions during assessments. To enhance reliability, testing procedures will be administered by the same investigator and all assessments will be done in the first hour of HT. Maximal isometric contraction force during extension of leg at knee joint, flexion and extension of lower limb at hip joint and abduction and adduction of lower limb at hip joint will be assessed by hand-held dynamometer (Universal digital force gauge HF 500, SAUTER GmbH, Balingen, Germany). During the assessments realized by the device, we will apply a customized pad that fits the shape of the lower extremity areas used for physical tests. The range of the dynamometer analyzer will be set from 0 to 500 N, with a recording interval of 0.1 N. An isometric contraction force assessments realized by a hand-held dynamometer showed an excellent reliability (IR ICC 0.940–0.963) and the measurement method showed a good validity compared with the results received from the isokinetic dynamometer (r = 0.806; *P* < .05).^[72]^

For an assessment, patients will be in a supine position with arms safely and comfortably placed on the bed. The measurements of the knee extension of the dominant leg will be done at a knee angle of 90° from full extension. The hand-held dynamometer will be placed on the patient's ankle and will be stabilized during the performance of the physical examination. During the assessments of hip flexion and extension of lower limb patient will hold the dominant leg in straightened position, while the dynamometer will be placed over the ankle (flexion force assessments) and under the heel (extension force assessments) of the measured leg. Lastly, the analysis of forces produced during hip abduction and adduction, will be done at a knee angle of 90° from full extension with dynamometer placed on the inner (adduction force assessments) and outer (abduction force assessments) side surface of knee joint. The patients will be instructed to perform a maximal isometric contraction and hold it for 5 seconds. The tests will be repeated within 30-second rest intervals, and the higher measured value of two consecutive tests will be used for the analysis.

#### 3.8.2 Secondary outcomes: patient's psycho-social variables

The changes in patient anxiety and depression states will be assessed by the Hospital Anxiety and Depression Scale.^[73]^ The scale consists of 2 subscales, 1 subscale for anxiety and another subscale for depression. Both subscales contain seven items and each item is rated on a 4-point scale (from 0 to 3 points). The maximal score for both subscales is 21, the minimum score is 0. Scores of 11 or more on either subscale are considered to be a significant indicator of psychological morbidity. Scores between 8 and 10 represent “borderline” and scores between 0 and 7 represents “normal” indication of anxiety and depression. The analyses of the scale's internal consistency in previous samples sizes showed a Cronbach's alpha varying between 0.82 and 0.90.^[74,75]^

The Kidney Disease and Quality of Life^[76,77]^ instrument will be used to assess the generic and kidney-disease specific quality of life of patients during the study. The instrument consists of a 36-item health survey, supplemented with 4 subscales targeted at particular concerns of individuals with kidney disease on dialysis (self-rated health, self-rated change of health status, a physical, and mental component). The analysis of the instrument's internal consistency showed a Cronbach's alpha varying between 0.84 and 0.91.^[78]^ All listed questionnaires and scales were translated, adapted and validated for use in the Slovak population by the methodology and standards described in procedural guidelines.^[79]^

#### 3.8.3 Secondary outcomes: patient's clinical data

Patient's clinical data (PCD) will be collected from the medical documentation of patients. During allocation, we will collect accessible socio-demographic data (age, gender) for all patients from medical database. The 1st PCD extraction will be assembled from the latest medical screening that was conducted prior to the start of the experimental or control condition in the study. The 2nd PCD extraction will be realized from the 1st medical screening realized after the completion of the experimental or control condition in the study. The collection of PCD will be completed also after the 3-month follow up period. Collected PCD will (apart from socio-demographic data) consist of: First, patient's nephrological diagnosis profile. Second, other present diagnosis and comorbidities. Third, body composition parameters (body weight and height, free fat mass and index, body fat mass assessed by Body composition monitoring^[80]^). Fourth, standard dialysis biochemistry, haematology, and dialysis adequacy measures.

#### 3.8.4 Secondary outcomes: miRNA analyses

The blood samples for miRNA analysis will be obtained during the regular dialysis session before the start of HT. In both study groups, the collection of blood samples from patients will be done 1 to 5 days prior to the start and after the completion of experimental and control condition. In the experimental group, blood samples for miRNA analysis will be also collected after the three-month follow up period. These samples will be analysed for the expression of specific, circulatory, calcium metabolism regulating miRNA's and for the expression of transcription factors involved in IGF-1 and TRIM63 signalling pathways. Also proteins encoded of these signalling pathways will be screened. The miRNA isolation and miRNA quantification will be realized following the protocol published by Blondal et al.^[81]^

### 3.9 Background variables

Background variables regard patient's life satisfaction, social support, health literacy, self-sufficiency, functional capacity and nutritional status.

The global cognitive judgments of a patient's life satisfaction will be measured by the Satisfaction with Life Scale.^[82]^ During the application of this scale, the patient will indicate how much he/she agrees or disagrees with each of the 5 items using a 7-point score. The mark of 7 points for an item indicates strong agreement, while lettering of 1 point is an expression of a strong disagreement with the item. The analyses of the scale's internal consistency in previous samples sizes showed a Cronbach's alpha varying between 0.89 and 0.90.^[83,84]^

Patient's social support will be measured by the Multidimensional Scale of Perceived Social Support.^[85]^ The scale contains three subscales, each represented by 4 questions addressing a different support source, all with a strong factorial validity in an analysis of social support of patient: A. Family, B. Friends, and C. Significant other sources. The analyses of the scale's internal consistency in previous samples sizes showed a Cronbach's alpha varying between 0.90 and 0.92.^[86,87]^

Patient's health literacy will be assessed using the Health Literacy Questionnaire,^[88,89]^ which enables us to effectively analyse a patient's personal, cognitive and social skills, which determine the ability of individuals to gain access to understand and use the information to promote and maintain his/her good health.^[90]^ The questionnaire is divided into 2 parts, which differ in response categories. Part 1 contains 5 scales with 4 response categories rating the extent of agreement from 1 to 4 (from 1-Strongly Disagree, to 4-Strongly Agree). Part 2 includes the last 4 scales with 5 response categories rating the level of difficulty from 1 to 5 (from 1-Cannot Do, to 5-Very Easy). The higher the average score in the scales indicates better health literacy. The analysis of the questionnaire's internal consistency in previous samples showed a Cronbach's alpha varying between 0.84 and 0.96.^[91]^

Patient's health literacy will further be assessed using the Brief Health Literacy survey^[92]^ is a short 3-item scale, where the answers are scored on a 5 point response scale (from 1 (Extremely) up to 5 (Not at all) points). It includes the following items: Firstly, How often do you have someone help you read hospital materials?, secondly, How confident are you filling out medical forms by yourself?, thirdly, How often do you have problems learning about your medical condition because of difficulty understanding written information? The item's response options are reversely scored and the higher total score given by the summation of scores in items indicates a higher subjective level of health literacy. The analyses of the survey's internal consistency in previous samples sizes showed a Cronbach's alpha varying between 0.72 and 0.80.^[93,94]^

The Karnofsky Performance Status^[95,96]^ scale will be used to assess the self-sufficiency and functional capacity of patients on HT. The scale determines functional impairment in the performance of activities of daily living regarding to scale rating criteria (ranging from 100 to 0). Scores of 80 or more on scale rating are considered to be an indicator of the ability to carry on normal activity and to work, with no need for special care. Scores between 79 and 50 regarding to scale rating criteria indicating an inability to work and ability to live at home and care for most personal needs with a varying amount of assistance. Scores lower than 50 pointing to an inability to care for self, requirements of institutional or hospital care and alerts that disease may be progressing rapidly. The three general scale definitions are supplemented with a 10-point analytical scale with more accurate identifications and definitions of patient's functional impairment rates. The analysis of the scale's internal consistency in a previous sample showed a Cronbach's alpha of 0.97.^[97]^

The nutritional status of patients will be assessed by the Subjective Global Assessment method.^[98]^ The method considers four features: 1. Patient's own nutritional history, 2. history of dietary intake in relation to a patient's usual pattern, 3. presence of significant gastrointestinal symptoms and 4. demands of the patient's underlying disease state. Based on these four features, the method indicates the patient category as well nourished, moderate or suspected malnutrition and severe malnutrition. The analysis of the method's internal consistency in a previous sample showed a Cronbach's alpha of 0.73.^[99]^

Translation, adaptation and validation of questionnaires and scales, for use in the Slovak population, was realized by the methodology and standards described in procedural guidelines.^[82]^ The subjective perceptions of changes after implementation of the IRT and knowledge about the limits and benefits of the intervention will be collected in the experimental group at the end of the experimental condition by the focus discussions and by the structured and semi-structured interviews. The subjective perceptions of changes after completion of control period will be also collected in the control group.

### 3.10 Procedures

All assessments, analyses and procedures will be realized exclusively by members of the study investigatory team. The assessments of the maximal voluntary contraction forces of lower extremity muscles and the collection of patient's clinical data will be realized in patients before and after the experimental or control condition and also after three months follow up period in both study groups by AZ and JR. The miRNA profiling will be performed before and after the experimental or control condition in both groups and also after the follow up period in the experimental group by MR. The assessments of the patient's quality of life and prevalence of anxiety and depression will be performed before and after the experimental and control group in both study groups by IS and PK. The determination and classification of patient's background variables will be realized only prior to the start of intervention or control period by IS, PK, AZ and JR. The assessments of patient's self-sufficiency, functional capacity and nutritional status will be also realized after the completion of experimental or control condition by AZ and JR. The subjective perceptions of changes after implementation of the IRT and knowledge about the limits and benefits of the intervention will be collected in the experimental group at the end of the experimental condition by focus discussions and semi-structured interviews by IS and AZ.

### 3.11 Data management and statistical analysis

All data will be deposed electronically in the project laptop. The laptop with confidential documents will be retained in the secured and monitored room in the Department of Health Psychology, Faculty of Medicine, Pavol Jozef Safarik University. In project databases, all patient's identification marks will be replaced by Euclid's algorithm number. To control the access to databases a password protection of the laptop will be used. Only authorized persons - all principal investigators - will have access to databases and evidence of study.

The project statistician (PK) will be responsible for data analyses and creation of the tables and figures containing mean, standard derivation, median, minimum and maximum values. The statistician will be blinded by the Euclid number coding of data sets. We will use the Shapiro–Wilk test for an assessment of the normality of data. To compare parametric variables between two groups we will used the Student *t* test. For paired parametric variables statistical analysis we will apply the paired t-test. In case of a non-parametric distribution of variables, we will analyse differences between 2 groups by the Wilcoxon Signed Rank test. For paired non-parametric variables we will process the data by the Mann–Whitney *U* test. For comparison of variables between 3 or more groups, we will apply a two-way ANOVA and MANOVA. Pearson chi-squared test will be utilized for statistical analysis of the categorical variables. The evidence of possible effects related to the experimental and control conditions will be conducted by estimated values and 95% confidence intervals.^[100]^ All data analyses will be carried out using the statistical software package IBM SPSS 22.0 or equivalent (IBM Corp. Released 2013, IBM SPSS Statistics for Windows, Version 22.0. Armonk, NY: IBM Corp.). All tests will be 2-tailed with statistical significance set at an α level of 0.05. The patients will be offered the possibility to receive the results of the study after completion of processing of data sets. In case of any modifications of the study protocol, the project representatives are responsible to report these changes in a trial registry (ClinicalTrails.gov) and to the Ethics Committee of the Louis Pasteur University Hospital in Kosice. All principal investigators will have access to the final study databases.

## 4 Discussion

Once the patient's size-adjusted estimated glomerular filtration rate is detected below 60 mL/min/1.73 m^2^ and/or markers of kidney damage were detectable for at least 3 months, she or he is reliant on kidney functions monitoring and management of progression and complications of CKD provided by a nephrologist.^[101]^ Further progression of chronic kidney disease is accompanied by the decision for initiation of the dialysis therapy. In these circumstances, the activation of HT is often a necessary step in maintaining the sufficient renal functions in patients. However, long term dialysis therapy setting is also the origin of a number of physiological and psycho-social adverse effects. Patients become more and more affected by regular exposure to prolonged inactivity and limitations in daily life. Evidence of several studies confirmed a high prevalence of sedentary behaviour, with physical activity and performance below the recommended levels among dialysed patients.^[102–104]^ The patients with CKD-5 suffered much more frequently and from much more severe depression and anxiety periods than healthy subjects did.^[26–28]^ Collectively, patients were affected by numerous factors that were interfering with their physical functions, psychological well-being and their quality of life.

Based on our recent experience and reviewed literature resources, we are expecting beneficial effects following the physical activity programme during HT. The significance will be proven by a range of improvements in lower extremity muscle functions. However, as we mentioned in the introduction, we are awaiting a high variability of patient's adaptability on IRT. The comparative analysis of high and low training responsiveness patients enables us to identify new conditional factors of the patient's adaptability during IRT in the clinical setting. We are expecting an identification of several psycho-social and clinical variables determining the effectiveness of IRT. Another relevant change in outcome confirming the utility of IRT is quality of life among patients with CKD-5. From the final results, it would be crucial to show a connection between functional, quality of life outcomes, and the prevalence of anxiety and depression.

A different source of new knowledge is associated with the analysis of circulatory miRNA profiles in patient groups. We are anticipating the identification of several miRNAs that strongly affect a calcification pathway and regulate or modify the functional adaptation during the IRT. The verification of IGF-1 and TRIM63 roles in calcium metabolism of patients with CKD-5 might forward the future research of novel diagnostic markers in clinical nephrology. Clarification of both metabolic pathways may add new knowledge in the area of the molecular physiology of functional adaptation processes following IRT. The activity of miRNAs involved in IGF-1 and TRIM63 metabolic pathways may also be identified as one of the effecting or modifying factors, explaining the individual adaptability of patients on IRT. A better understanding of changes in gene expression during dialysis and exercise intervention can be used for many purposes, like the detection of progressive pathological changes of the renal system, for the experimental study of receptor inhibitors/activators against muscle function and mass loss or receptor functions during trials targeted on slowing down patient's cachectic obesity.^[105]^

The decision to apply a quasi-experimental design originates from the logistics of study. The demands for close control and evidence of the IRT implementation by experienced assistants and the demands of personal capacities and qualities for management of the IRT programme forced us to use a not-randomized design in our study. At the point of design finalization, we recognized that the allocation of patients into the experimental and control group might be a source of bias during comparisons of outcomes between the experimental and control group. To avoid this threat of internal validity, we included three nephrology clinics with identical care-providing standards and with similar numbers of patients eligible for the study.

## Acknowledgments

We appreciate the co-operation of the representatives and staff of dialysis centres involved in the implementation of this study. Special acknowledgements go to Peter Mizla MD and Peter Javorsky MD for organizational support during study planning and implementation.

## Author contributions

JPvD, SAR, JR, MR, MM, PK, AMG, and AZ created the idea of the study and critically revised the protocol of the study. IS, PK, AZ, and the NEPHRO-team drafted the manuscript. PK provided statistical analysis plan for manuscript and will performed the data analysis in the study. MR provided description of miRNA analysis methods in manuscript and will be responsible for miRNA analyses during implementation of protocol. IS, PK, JR, MR, AZ, and the NEPHRO-team will be responsible for implementation of the study protocol in the dialysis centres. All authors read and approved the final manuscript.

**Conceptualization:** Aurel Zelko, Peter Kolarcik, Jaroslav Rosenberger, Miroslava Rabajdova, Maria Marekova, Andrea Madarasova Geckova, Jitse P. van Dijk, Sijmen A. Reijneveld.

**Data curation:** Aurel Zelko, Ivana Skoumalova, Peter Kolarcik, Miroslava Rabajdova, Maria Marekova, Andrea Madarasova Geckova, NEPHRO team.

**Formal analysis:** Aurel Zelko, Jaroslav Rosenberger, Miroslava Rabajdova, Maria Marekova, Andrea Madarasova Geckova, Jitse P. van Dijk, Sijmen A. Reijneveld, NEPHRO team.

**Funding acquisition:** Aurel Zelko, Ivana Skoumalova, Peter Kolarcik, Jaroslav Rosenberger.

**Investigation:** Aurel Zelko, Ivana Skoumalova, Peter Kolarcik, Jaroslav Rosenberger, Miroslava Rabajdova, Maria Marekova, Andrea Madarasova Geckova, NEPHRO team.

**Methodology:** Aurel Zelko, Ivana Skoumalova, Peter Kolarcik, Jaroslav Rosenberger, Miroslava Rabajdova, Maria Marekova, Andrea Madarasova Geckova, Jitse P. van Dijk, Sijmen A. Reijneveld, NEPHRO team.

**Project administration:** Aurel Zelko, Ivana Skoumalova, Peter Kolarcik, Jaroslav Rosenberger, Miroslava Rabajdova, Andrea Madarasova Geckova, NEPHRO team.

**Supervision:** Aurel Zelko, Peter Kolarcik, Jaroslav Rosenberger, Miroslava Rabajdova, Maria Marekova, Andrea Madarasova Geckova, Jitse P. van Dijk, Sijmen A. Reijneveld.

**Validation:** Aurel Zelko, Ivana Skoumalova, Peter Kolarcik, Jaroslav Rosenberger, Miroslava Rabajdova, Maria Marekova, Andrea Madarasova Geckova, Jitse P. van Dijk, Sijmen A. Reijneveld, NEPHRO team.

**Writing – original draft:** Aurel Zelko, Andrea Madarasova Geckova, Jitse P. van Dijk, Sijmen A. Reijneveld.

**Writing – review & editing:** Aurel Zelko, Ivana Skoumalova, Peter Kolarcik, Jaroslav Rosenberger, Miroslava Rabajdova, Maria Marekova, Andrea Madarasova Geckova, Jitse P. van Dijk, Sijmen A. Reijneveld, NEPHRO team.

Aurel Zelko orcid: 0000-0001-9682-2225.

## References

[1] Liyanage T Ninomiya T Jha V Worldwide access to treatment for end-stage kidney disease: a systematic review. Lancet 2015;385:1975–82. 25777665 10.1016/S0140-6736(14)61601-9

[2] Rechel B Grundy E Robine JM Ageing in the European Union. Lancet 2013;381:1312–22. 23541057 10.1016/S0140-6736(12)62087-X

[3] Shaw JE Sicree RA Zimmet PZ Global estimates of the prevalence of diabetes for 2010 and 2030. Diabetes Res Clin Pract 2010;87:4–14. 19896746 10.1016/j.diabres.2009.10.007

[4] Kelly T Yang W Chen CS Global burden of obesity in 2005 and projections to 2030. Int J Obes (Lond) 2008;32:1431–7. 18607383 10.1038/ijo.2008.102

[5] National Kidney Foundation. KDOQI Clinical practice guideline for hemodialysis adequacy: 2015 update. Am J Kidney Dis 2015;66:884–930. 26498416 10.1053/j.ajkd.2015.07.015

[6] Neild GH Life expectancy with chronic kidney disease: an educational review. Pediatr Nephrol 2017;32:243–8. 27115888 10.1007/s00467-016-3383-8 PMC5203814

[7] Kao TW Huang JW Hung KY Life expectancy, expected years of life lost and survival of hemodialysis and peritoneal dialysis patients. J Nephrol 2010;23:677–82. 20540032

[8] Alfaadhel TA Soroka SD Kiberd BA Frailty and mortality in dialysis: evaluation of a clinical frailty scale. Clin J Am Soc Nephrol 2015;10:832–40. 25739851 10.2215/CJN.07760814 PMC4422241

[9] Bao Y Dalrymple L Chertow GM Frailty, dialysis initiation and mortality in end-stage renal disease. Arch Intern Med 2012;172:1071–7. 22733312 10.1001/archinternmed.2012.3020 PMC4117243

[10] McAdams-DeMarco MA Law A Salter ML Frailty as a novel predictor of mortality and hospitalization in individuals of all ages undergoing hemodialysis. J Am Geriatr Soc 2013;61:896–901. 23711111 10.1111/jgs.12266 PMC3938084

[11] McIntyre CW Selby NM Sigrist M Patients receiving maintenance dialysis have more severe functionally significant skeletal muscle wasting than patients with dialysis-independent chronic kidney disease. Nephrol Dial Transplant 2006;21:2210–6. 16504974 10.1093/ndt/gfl064

[12] John SG Sigrist MK Taal MW Natural history of skeletal muscle mass changes in chronic kidney disease stage 4 and 5 patients: an observational study. PLoS One 2013;8:e65372. 23741490 10.1371/journal.pone.0065372 PMC3669290

[13] Sutcliffe BK Bennett PN Fraser SF The deterioration in physical function of hemodialysis patients. Hemodial Int 2018;22:245–53. 28474859 10.1111/hdi.12570

[14] Johansen KL Shubert T Doyle J Muscle atrophy in patients receiving hemodialysis: effects on muscle strength, muscle quality, and physical function. Kidney Int 2003;63:291–7. 12472795 10.1046/j.1523-1755.2003.00704.x

[15] Wang AY Sherrington C Toyama T Muscle strength, mobility, quality of life and falls in patients on maintenance haemodialysis: a prospective study. Nephrology (Carlton) 2017;22:220–7. 26890468 10.1111/nep.12749

[16] Oller GA RibeiroRde C Travagim DS Functional independence in patients with chronic kidney disease being treated with haemodialysis. Rev Lat Am Enfermagem 2012;20:1033–40. 23258715 10.1590/s0104-11692012000600004

[17] Cheema B Abas H Smith B Investigation of skeletal muscle quantity and quality in end-stage renal disease. Nephrology (Carlton) 2010;15:454–63. 20609098 10.1111/j.1440-1797.2009.01261.x

[18] Rasić-Milutinović Z Perunicić-Peković G Ristić-Medić D Insulin resistance and chronic inflammation are associated with muscle wasting in end-stage renal disease patients on hemodialysis. Gen Physiol Biophys 2009;28: 19893099

[19] Moreau-Gaudry X Jean G Genet L A simple protein-energy wasting score predicts survival in maintenance hemodialysis patients. J Ren Nutr 2014;24:395–400. 25194620 10.1053/j.jrn.2014.06.008

[20] Windahl K Faxén Irving G Almquist T Prevalence and risk of protein-energy wasting assessed by subjective global assessment in older adults with advanced chronic kidney disease: results from the EQUAL Study. J Ren Nutr 2018;28:165–74. 29459026 10.1053/j.jrn.2017.11.002

[21] van der Ploeg HP Chey T Korda RJ Sitting time and all-cause mortality risk in 222 497 Australian adults. Arch Intern Med 2012;172:494–500. 22450936 10.1001/archinternmed.2011.2174

[22] Manns P In people aged over 45, increased time spent sitting daily is associated with increased risk of all-cause mortality independent of physical activity level. Evid Based Nurs 2012;15:120–1. 22864524 10.1136/eb-2012-100843

[23] Lee IM Shiroma EJ Lobelo F Lancet Physical Activity Series Working Group. Effect of physical inactivity on major non-communicable diseases worldwide: an analysis of burden of disease and life expectancy. Lancet 2012;380:219–29. 22818936 10.1016/S0140-6736(12)61031-9 PMC3645500

[24] Olvera-Soto MG Valdez-Ortiz R López Alvarenga JC Effect of resistance exercises on the indicators of muscle reserves and handgrip strength in adult patients on hemodialysis. J Ren Nutr 2016;26:53–60. 26264173 10.1053/j.jrn.2015.06.006

[25] Molsted S Bjørkman ASD Lundstrøm LH Effects of strength training to patients undergoing dialysis: a systematic review. Dan Med J 2019;66 (1.):ii:A5526. 30573007

[26] Al-Jabi SW Sous A Jorf F Depression in patients treated with haemodialysis: a cross-sectional study. Lancet 2018;391 Suppl2:S41. 29553441 10.1016/S0140-6736(18)30407-0

[27] Lopes AA Albert JM Young EW Screening for depression in hemodialysis patients: associations with diagnosis, treatment, and outcomes in the DOPPS. Kidney Int 2004;66:2047–53. 15496178 10.1111/j.1523-1755.2004.00977.x

[28] Kopple JD Shapiro BB Feroze U Hemodialysis treatment engenders anxiety and emotional distress. Clin Nephrol 2017;88:205–17. 28818189 10.5414/CN109112

[29] Cukor D Coplan J Brown C Anxiety disorders in adults treated by hemodialysis: a single-center study. Am J Kidney Dis 2008;52:128–36. 18440682 10.1053/j.ajkd.2008.02.300

[30] Zamanian H Poorolajal J Taheri-Kharameh Z Relationship between stress coping strategies, psychological distress, and quality of life among hemodialysis patients. Perspect Psychiatr Care 2018;54:410–5. 29689625 10.1111/ppc.12284

[31] Hiles SA Lamers F Milaneschi Y Sit, step, sweat: longitudinal associations between physical activity patterns, anxiety and depression. Psychol Med 2017;47:1466–77. 28137333 10.1017/S0033291716003548

[32] Helgadóttir B Forsell Y Ekblom Ö Physical activity patterns of people affected by depressive and anxiety disorders as measured by accelerometers: a cross-sectional study. PLoS One 2015;10:e0115894. 25585123 10.1371/journal.pone.0115894 PMC4293141

[33] Zhang M Kim JC Li Y Relation between anxiety, depression, and physical activity and performance in maintenance hemodialysis patients. J Ren Nutr 2014;24:252–60. 24788308 10.1053/j.jrn.2014.03.002 PMC4103694

[34] Dziubek W Kowalska J Kusztal M The level of anxiety and depression in dialysis patients undertaking regular physical exercise training - a preliminary study. Kidney Blood Press Res 2016;41:86–98. 26872253 10.1159/000368548

[35] Frih B Mkacher W Bouzguenda A Effects of listening to Holy Qur’an recitation and physical training on dialysis efficacy, functional capacity, and psychosocial outcomes in elderly patients undergoing haemodialysis. Libyan J Med 2017;12:1372032. 28891419 10.1080/19932820.2017.1372032 PMC5650043

[36] Chung YC Yeh ML Liu YM Effects of intradialytic exercise on the physical function, depression and quality of life for haemodialysis patients: a systematic review and meta-analysis of randomised controlled trials. J Clin Nurs 2017;26:1801–13. 27532211 10.1111/jocn.13514

[37] Anding K Bär T Trojniak-Hennig J A structured exercise programme during haemodialysis for patients with chronic kidney disease: clinical benefit and long-term adherence. BMJ Open 2015;5:e008709. 10.1136/bmjopen-2015-008709 PMC4554901 26316654

[38] Zhang L Luo H Kang G The association between physical activity and mortality among patients undergoing maintenance hemodialysis. Int J Nurs Pract 2017;23: 10.1111/ijn.12505 28026071

[39] Shimoda T Matsuzawa R Yoneki K Changes in physical activity and risk of all-cause mortality in patients on maintence hemodialysis: a retrospective cohort study. BMC Nephrol 2017;18:154. 28482880 10.1186/s12882-017-0569-7 PMC5422904

[40] Stack AG Molony DA Rives T Association of physical activity with mortality in the US dialysis population. Am J Kidney Dis 2005;45:690–701. 15806472 10.1053/j.ajkd.2004.12.013

[41] Inaguma D Tanaka A Shinjo H Physical function at the time of dialysis initiation is associated with subsequent mortality. Clin Exp Nephrol 2017;21:425–35. 27392911 10.1007/s10157-016-1307-3

[42] Liao MT Liu WC Lin FH Intradialytic aerobic cycling exercise alleviates inflammation and improves endothelial progenitor cell count and bone density in hemodialysis patients. Medicine (Baltimore) 2016;95:e4134. 27399127 10.1097/MD.0000000000004134 PMC5058856

[43] Moraes C Marinho SM da Nobrega AC Resistance exercise: a strategy to attenuate inflammation and protein-energy wasting in hemodialysis patients? Int Urol Nephrol 2014;46:1655–62. 24729104 10.1007/s11255-014-0712-3

[44] Paluchamy T Vaidyanathan R Effectiveness of intradialytic exercise on dialysis adequacy, physiological parameters, biochemical markers and quality of life – a pilot study. Saudi J Kidney Dis Transpl 2018;29:902–10. 30152428 10.4103/1319-2442.239661

[45] Toyama K Sugiyama S Oka H Exercise therapy correlates with improving renal function through modifying lipid metabolism in patients with cardiovascular disease and chronic kidney disease. J Cardiol 2010;56:142–6. 20696551 10.1016/j.jjcc.2010.06.007

[46] Castaneda C Gordon PL Uhlin KL Resistance training to counteract the catabolism of a low-protein diet in patients with chronic renal insufficiency. A randomized, controlled trial. Ann Intern Med 2001;135:965–76. 11730397 10.7326/0003-4819-135-11-200112040-00008

[47] Orcy R Antunes MF Schiller T Aerobic exercise increases phosphate removal during hemodialysis: a controlled trial. Hemodial Int 2014;18:450–8. 24438516 10.1111/hdi.12123

[48] Baria F Kamimura MA Aoike DT Randomized controlled trial to evaluate the impact of aerobic exercise on visceral fat in overweight chronic kidney disease patients. Nephrol Dial Transplant 2014;29:857–64. 24449105 10.1093/ndt/gft529

[49] Frih B Jaafar H Mkacher W The effect of interdialytic combined resistance and aerobic exercise training on health related outcomes in chronic hemodialysis patients: the Tunisian randomized controlled study. Front Physiol 2017;8:288. 28620308 10.3389/fphys.2017.00288 PMC5449721

[50] Parsons TL Toffelmire EB King-VanVlack CE Exercise training during hemodialysis improves dialysis efficacy and physical performance. Arch Phys Med Rehabil 2006;87:680–7. 16635631 10.1016/j.apmr.2005.12.044

[51] Musavian AS Soleimani A MasoudiAlavi N Comparing the effects of active and passive intradialytic pedaling exercises on dialysis efficacy, electrolytes, hemoglobin, hematocrit, blood pressure and health-related quality of life. Nurs Midwifery Stud 2015;4:e25922. 25830161 10.17795/nmsjournal25922 PMC4377533

[52] Afshar R Shegarfy L Shavandi N Effects of aerobic exercise and resistance training on lipid profiles and inflammation status in patients on maintenance hemodialysis. Indian J Nephrol 2010;20:185–9. 21206679 10.4103/0971-4065.73442 PMC3008946

[53] Leehey DJ Moinuddin I Bast JP Aerobic exercise in obese diabetic patients with chronic kidney disease: a randomized and controlled pilot study. Cardiovasc Diabetol 2009;8:62. 20003224 10.1186/1475-2840-8-62 PMC2796994

[54] Headley S Germain M Milch C Exercise training improves HR responses and VO2peak in predialysis kidney patients. Med Sci Sports Exerc 2012;44:2392–9. 22811032 10.1249/MSS.0b013e318268c70c

[55] Chigira Y Oda T Izumi M Effects of exercise therapy during dialysis for elderly patients undergoing maintenance dialysis. J Phys Ther Sci 2017;29:20–3. 28210031 10.1589/jpts.29.20 PMC5300797

[56] D'Souza RF Markworth JF Aasen KMM Acute resistance exercise modulates microRNA expression profiles: Combined tissue and circulatory targeted analyses. PLoS One 2017;12:e0181594. 28750051 10.1371/journal.pone.0181594 PMC5531502

[57] Uhlemann M Möbius-Winkler S Fikenzer S Circulating microRNA-126 increases after different forms of endurance exercise in healthy adults. Eur J PrevCardiol 2014;21:484–91. 10.1177/2047487312467902 23150891

[58] Horak M Zlamal F Iliev R Exercise-induced circulating microRNA changes in athletes in various training scenarios. PLoS One 2018;13:e0191060. 29338015 10.1371/journal.pone.0191060 PMC5770042

[59] Baggish AL Hale A Weiner RB Dynamic regulation of circulating microRNA during acute exhaustive exercise and sustained aerobic exercise training. J Physiol 2011;589 (Pt 16):3983–94. 21690193 10.1113/jphysiol.2011.213363 PMC3179997

[60] Bye A Røsjø H Aspenes ST Circulating microRNAs and aerobic fitness--the HUNT-Study. PLoS One 2013;8:e57496. 23469005 10.1371/journal.pone.0057496 PMC3585333

[61] Van Craenenbroeck AH Ledeganck KJ Van Ackeren K Plasma levels of microRNA in chronic kidney disease: patterns in acute and chronic exercise. Am J Physiol Heart Circ Physiol 2015;309:H2008–16. 26475583 10.1152/ajpheart.00346.2015 PMC4698424

[62] Chan AW Tetzlaff JM Altman DG SPIRIT 2013 Statement: defining standard protocol items for clinical trials. Ann Intern Med 2013;158:200–7. 23295957 10.7326/0003-4819-158-3-201302050-00583 PMC5114123

[63] Chan A-W Tetzlaff JM Gøtzsche PC SPIRIT 2013 explanation and elaboration: guidance for protocols of clinical trials. BMJ 2013;346:e7586. 23303884 10.1136/bmj.e7586 PMC3541470

[64] Charlson ME Pompei P Ales KL A new method of classifying prognostic comorbidity in longitudinal studies: development and validation. J Chronic Dis 1987;40:373–83. 3558716 10.1016/0021-9681(87)90171-8

[65] Molsted S Harrison AP Eidemak I The effects of high-load strength training with protein- or non protein- containing nutritional supplementation in patients undergoing dialysis. J Ren Nutr 2013;23:132–40. 22959782 10.1053/j.jrn.2012.06.007

[66] Kirkman DL Mullins P Junglee NA Anabolic exercise in haemodialysis patients: a randomised controlled pilot study. J Cachexia Sarcopenia Muscle 2014;5:199–207. 24710697 10.1007/s13539-014-0140-3 PMC4159488

[67] Cheema B Abas H Smith B Progressive exercise for anabolism in kidney disease (PEAK): a randomized, controlled trial of resistance training during hemodialysis. J Am Soc Nephrol 2007;18:1594–601. 17409306 10.1681/ASN.2006121329

[68] Bennett PN Daly RM Fraser SF The impact of an exercise physiologist coordinated resistance exercise program on the physical function of people receiving hemodialysis: a stepped wedge randomised control study. BMC Nephrol 2013;14:204. 24070232 10.1186/1471-2369-14-204 PMC3850647

[69] Esgalhado M Stockler-Pinto MB de França Cardozo LF Effect of acute intradialytic strength physical exercise on oxidative stress and inflammatory responses in hemodialysis patients. Kidney Res Clin Pract 2015;34:35–40. 26484017 10.1016/j.krcp.2015.02.004 PMC4570601

[70] Smart NA Williams AD Levinger I Exercise & Sports Science Australia (ESSA) position statement on exercise and chronic kidney disease. J Sci Med Sport 2013;16:406–11. 23434075 10.1016/j.jsams.2013.01.005

[71] Matsuzawa R Matsunaga A Wang G Relationship between lower extremity muscle strength and all-cause mortality in Japanese patients undergoing dialysis. Phys Ther 2014;94:947–56. 24578522 10.2522/ptj.20130270

[72] Kim WK Kim DK Seo KM Reliability and validity of isometric knee extensor strength test with hand-held dynamometer depending on its fixation: a pilot study. Ann Rehabil Med 2014;38:84–93. 24639931 10.5535/arm.2014.38.1.84 PMC3953369

[73] Zigmond AS Snaith P The hospital anxiety and depression scale. Acta Psychiatr Scand 1983;67:361–70. 6880820 10.1111/j.1600-0447.1983.tb09716.x

[74] Wang W Chair SY Thompson DR A psychometric evaluation of the Chinese version of the Hospital Anxiety and Depression Scale in patients with coronary heart disease. J Clin Nurs 2009;18:2436–43. 19694877 10.1111/j.1365-2702.2009.02807.x

[75] Sukantarat KT Williamson RC Brett SJ Psychological assessment of ICU survivors: a comparison between the Hospital Anxiety and Depression scale and the Depression, Anxiety and Stress scale. Anaesthesia 2007;62:239–43. 17300300 10.1111/j.1365-2044.2006.04948.x

[76] Hays RD Kallich JD Mapes DL Development of the Kidney Disease Quality of Life (KDQOL) instrument. Qual Life Res 1994;3:329–38. 7841967 10.1007/BF00451725

[77] Hays RD Kallich JD Mapes DL Kidney Disease Quality of Life Short Form (KDQOL-SF). A manual for use and scoring. Ver. 1.3. Santa Monica: RAND 1997 7994.

[78] Yildirim A Ogutmen B Bektas G Translation, cultural adaptation, initial reliability, and validation of the Kidney Disease and Quality of Life-Short Form (KDQOL-SF 1.3) in Turkey. Transplant Proc 2007;39:51–4. 17275473 10.1016/j.transproceed.2006.10.196

[79] Gudmundsson E Guidelines for translating and adapting psychological instruments. Nordic Psychol 2009;61:29–45.

[80] Rosenberger J Kissova V Majernikova M Body composition monitor assessing malnutrition in the hemodialysis population independently predicts mortality. J Ren Nutr 2014;24:172–6. 24618132 10.1053/j.jrn.2014.01.002

[81] Blondal T Jensby Nielsen S Baker A Assessing sample and miRNA profile quality in serum and plasma or other biofluids. Methods 2013;59:S1–6. 23036329 10.1016/j.ymeth.2012.09.015

[82] Diener E Emmons RA Larsen RJ The satisfaction with life scale. J Pers Assess 1985;49:71–5. 16367493 10.1207/s15327752jpa4901_13

[83] Maroufizadeh S Ghaheri A Omani Samani R Psychometric properties of the satisfaction with life scale (SWLS) in Iranian infertile women. Int J Reprod Biomed (Yazd) 2016;14:57–62. 27141550 PMC4837918

[84] Rosengren L Jonasson SB Brogårdh C Psychometric properties of the satisfaction with life scale in Parkinson's disease. Acta Neurol Scand 2015;132:164–70. 25639961 10.1111/ane.12380

[85] Zimet GD Dahlem NW Zimet SG The multidimensional scale of perceived social support. J Pers Assess 1988;52:30–41.

[86] Vaingankar JA Abdin E Chong SA Exploratory and confirmatory factor analyses of the Multidimensional Scale of Perceived Social Support in patients with schizophrenia. Compr Psychiatry 2012;53:286–91. 21632040 10.1016/j.comppsych.2011.04.005

[87] Akhtar A Rahman A Husain M Multidimensional scale of perceived social support: psychometric properties in a South Asian population. J Obstet Gynaecol Res 2010;36:845–51. 20666955 10.1111/j.1447-0756.2010.01204.x

[88] Osborne RH Batterham RW Elsworth GR The grounded psychometric development and initial validation of the Health Literacy Questionnaire (HLQ). BMC Public Health 2013;13:658. 23855504 10.1186/1471-2458-13-658 PMC3718659

[89] Kolarcik P Cepova E Madarasova Geckova A Structural properties and psychometric improvements of the Health Literacy Questionnaire in a Slovak population. Int J Public Health 2017;62:591–604. 28258403 10.1007/s00038-017-0945-x

[90] Nutbeam D Health promotion glossary. Health Promot 1986;1:113–27. 10318625 10.1093/heapro/1.1.113

[91] Haghdoost AA Rakhshani F Aarabi M Iranian Health Literacy Questionnaire (IHLQ): an instrument for measuring health literacy in Iran. Iran Red Crescent Med J 2015;17:e25831. 26290752 10.5812/ircmj.17(5)2015.25831 PMC4537788

[92] Chew LD Bradley KA Boyko EJ Brief questions to identify patients within adequate health literacy. Fam Med 2004;36:588–94. 15343421

[93] Wallston KA Cawthon C McNaughton CD Psychometric properties of the brief health literacy screen in clinical practice. J Gen Intern Med 2014;29:119–26. 23918160 10.1007/s11606-013-2568-0 PMC3889960

[94] Cavanaugh KL Osborn CY Tentori F Performance of a brief survey to assess health literacy in patients receiving hemodialysis. Clin Kidney J 2015;8:462–8. 26251719 10.1093/ckj/sfv037 PMC4515892

[95] Karnofsky DA Burchenal JH The clinical evaluation of chemotherapeutic agents in cancer. In: Macleod C.M., (ed.), Evaluation of Chemotherapeutic Agents. New York, NY, USA: Columbia University Press; 1949.

[96] Crooks V Waller S Smith T The use of the Karnofsky Performance Scale in determining outcomes and risk in geriatric outpatients. J Gerontol 1991;46:M139–44. 2071835 10.1093/geronj/46.4.m139

[97] Mor V Laliberte L Morris JN The Karnofsky Performance Status Scale. An examination of its reliability and validity in a research setting. Cancer 1984;53:2002–7. 6704925 10.1002/1097-0142(19840501)53:9<2002::aid-cncr2820530933>3.0.co;2-w

[98] Detsky AS McLaughlin JR Baker JP What is subjective global assessment of nutritional status? JPEN J Parenter Enteral Nutr 1987;11:8–13. 3820522 10.1177/014860718701100108

[99] Campbell KL Ash S Bauer J Critical review of nutrition assessment tools to measure malnutrition in chronic kidney disease. Nutr Diet 2007;64 (1.):23–30. 10.1053/j.jrn.2006.12.005 17462551

[100] Armitage P Berry G Matthews JNS Statistical methods in medical research. 4th edition Oxford: Blackwell Science; 2001.

[101] Improving Global Outcomes (KDIGO) CKD Work Group. Summary of recommendation statements. In: KDIGO 2012 clinical practice guideline for the evaluation and management of chronic kidney disease. Kidney Int Suppl (2011) 2013;3:5–14.25598998

[102] Akber A Portale AA Johansen KL Pedometer-assessed physical activity in children and young adults with CKD. Clin J Am Soc Nephrol 2012;7:720–6. 22422539 10.2215/CJN.06330611 PMC3338276

[103] Cupisti A D’Alessandro C Finato V Assessment of physical activity, capacity and nutritional status in elderly peritoneal dialysis patients. BMC Nephrol 2017;18:180. 28558794 10.1186/s12882-017-0593-7 PMC5450102

[104] Cobo G Gallar P Gama-Axelsson T Clinical determinants of reduced physical activity in hemodialysis and peritoneal dialysis patients. J Nephrol 2015;28:503–10. 25501981 10.1007/s40620-014-0164-y

[105] Rabajdová M Urban P Špaková I Detection of pathological changes in the aorta during thoracic aortic aneurysm progression on molecular level. Dis Markers 2017;2017:9185934. 29158612 10.1155/2017/9185934 PMC5660829

